# Innovation in Systems of Care in Acute Phase of Ischemic Stroke. The Experience of the Catalan Stroke Programme

**DOI:** 10.3389/fneur.2018.00427

**Published:** 2018-06-06

**Authors:** Rosa M. Vivanco-Hidalgo, Sònia Abilleira, Mercè Salvat-Plana, Aida Ribera, Guillem Gallofré, Miquel Gallofré

**Affiliations:** ^1^Stroke Programme, Catalan Stroke Foundation, Barcelona, Spain; ^2^Stroke Programme, Agency for Health Quality and Assessment of Catalonia, CIBER Epidemiología y Salud Pública, Barcelona, Spain; ^3^Stroke Programme, Barcelona, Spain; ^4^Cardiovascular Epidemiology Unit, Cardiology Department, Hospital Vall d'Hebron, Barcelona, Spain

**Keywords:** stroke, systems of care, innovation, endovascular treatment of stroke, thrombolysis

## Abstract

Stroke, and mainly ischemic stroke, is the second cause of death and disability. To confront the huge burden of this disease, innovative stroke systems of care are mandatory. This requires the development of national stroke plans to offer the best treatment to all patients eligible for reperfusion therapies. Key elements for success include a high level of organization, close cooperation with emergency medical services for prehospital assessment, an understanding of stroke singularity, the development of preassessment tools, a high level of commitment of all stroke teams at Stroke Centres, the availability of a disease-specific registry, and local government involvement to establish stroke care as a priority. In this mini review, we discuss recent evidence concerning different aspects of stroke systems of care and describe the success of the Catalan Stroke Programme as an example of innovation. In Catalonia, reperfusion treatment rates have increased in recent years and currently are among the highest in Europe (17.3% overall, 14.3% for IVT, and 6% for EVT in 2016).

## Introduction

Stroke—mainly ischemic stroke—is the second cause of death and disability worldwide (1). The stroke burden has increased across the globe in both men and women of all ages throughout the past two decades (2). However, population awareness of early symptoms, the accuracy of current brain imaging tests, and the development of acute therapies are contributing to reduce this trend (3). However, success will depend mainly on the structure of the healthcare system and it is uncertain whether systems in different countries are prepared to deal with this huge burden. Therefore, innovation in stroke systems of care is mandatory to transform them and prepare them to confront this health challenge.

## Innovating in systems of acute stroke care. organization and cooperation to deliver more treatment, more rapidly

The natural history of ischaemic stroke has changed dramatically since the 1990s. The beginning of the Intravenous Thrombolysis (IVT) Era in the late 90s (4) and recent approval of endovascular therapy (EVT) (5), the demonstration of improved stroke outcomes with stroke unit care, and the benefits of implementing organized stroke systems of care have all contributed to reduce mortality and disability in patients with acute ischaemic stroke (AIS) (3, 6).

The proportion of patients treated has increased in recent years in high-income countries, mainly in comprehensive stroke centers (CSCs), where EVT is provided (7). The effectiveness of reperfusion therapies is highly time-dependent: The sooner the patient arrives to the hospital after symptoms onset, the better. Once in hospital, highly organized workflows are of utmost importance to achieve door-to-needle times in IVT and door-to-groin-puncture times in EVT. A set of effective strategies has been successfully implemented in hospitals to reduce these critical time delays, especially pre-notification of arrival by Emergency Medical Services (EMS), direct transfer to the radiology service for brain CT scan or direct alteplase administration in the scanner (8, 9).

The eligibility of patients with AIS for reperfusion therapies is evolving as new evidence is published. Partially dependent patients, otherwise excluded from IVT trials, might benefit from thrombolysis (10) and certain patients with unknown time of stroke onset, precluded from seminal EVT trials, have shown improved outcomes after EVT (11, 12). Therefore, the overall proportion of AIS patients who might benefit from reperfusion therapies is rising and changing inclusion criteria generate uncertainty about how many more are potentially eligible.

The capacity to increase the proportion of patients treated is related to the structure of the system of care. Current evidence shows that patients who require interhospital transfer for EVT achieve reperfusion between 109 and 120 min later than those directly transported to CSCs, and have a lower absolute probability of independent outcome (13–15). These data should prompt a rethinking of systems of stroke care at national and regional levels in order to improve the “hub-and-spoke” transfer networks. This form of medical transport optimization organizes traffic routes as a series of 'spokes' that connect outlying points to a central 'hub.' In acute care for stroke, the Hubs are CSCs with great expertise that concentrate a huge volume of procedures and are connected with centers having a lower level of expertise and smaller volume of procedures.

## Understanding the singularity of stroke to improve care

Management of AIS has taken lessons from acute myocardial infarction management; however, unlike heart attack, in most cases acute stroke leaves patients unable to speak and to alert EMS by themselves. Population campaigns are crucial to raise awareness about stroke symptoms and how to detect them. In addition, EMS technicians must be specifically trained to detect stroke, activate the stroke code and pre-notify arrival to the nearest hospital with proper treatment capabilities.

The possibility of diagnostic testing in the ambulance to inform hospital treatment is another huge difference between heart attack and stroke. The recent development of CT-equipped mobile stroke units is considered an important advancement. This approach is safe and feasible, has increased IVT rates, and achieved significantly shorter time-to-treatment compared to conventional care in the areas tested, mainly in Germany (16). However, a recent study found no significant difference between the proportion of patients with a modified Rankin Scale score of 1 or less who received this type of care compared with conventional care (17). Therefore, due to its high cost without clear long-term benefit, we can conclude that an efficient technology for prehospital diagnosis of stroke that can be easily implemented is lacking.

## Organizing to achieve better results. organization and cooperation at different levels of care

Once the stroke code is activated, where do we transfer the patient? Given the beneficial results of bridging therapy (IVT plus EVT) in patients with large vessel occlusion (LVO) (5), it seems clear that the demand for neurointervention in coming years will grow in line with increasing numbers of EVT-capable centers. Nonetheless, it is difficult to justify the establishment of EVT-capable centers in remote areas with low population density.

Therefore, a crucial question is how to define the best transfer network for AIS patients located in remote and distant areas. The drip-and-ship model, which takes the patient to the nearest stroke center, prioritizes the initial diagnostic workup and IVT. In this model, the identification of an LVO patient is followed by interhospital transfer to a CSC. Another model is direct transfer to a CSC, thus bypassing the Primary Stroke Centre (PSC), known as the mothership model. A recent study in Canada used conditional probability modeling to find an answer, testing different transportation options to identify the better modeled outcome in specific regions. The authors concluded that a drip-and-ship model is appropriate if the treatment in a PSC is delivered in less than 30 min and the patient is then transferred to a capable CSC (18). In Catalonia, the ongoing RACECAT trial (Direct Transfer to an Endovascular Centre Compared to Transfer to the Closest Stroke Centre in Acute Stroke Patients with Suspected Large Vessel Occlusion; NCT02795962) is expected to provide answers to important questions of logistics and increase the efficient delivery of treatments and the number of acute stroke patients that have access to them.

There is still room for network innovation. In remote areas with no access to stroke experts, a possible and feasible solution is TeleStroke Centres (TSC). Using videoconferencing and image-sharing technology, stroke specialists from a CSC can examine patients at remote hospitals to help with diagnosis and recommend a plan of care.

Results from a third model, called trip-and-treat, have been recently published. This urban interhospital service delivery model consists of a shared mobile interventional stroke team that travels to PSCs in New York City to provide on-site interventional capability. The authors concluded that, in their area of reference, the trip-and-treat model had shorter time-to-treatment for EVT, compared with drip-and-ship, offering a valid alternative to current interhospital stroke transfers in urban environments (19).

## Prehospital assessment

Theoretically, the benefits of a primary transfer to a CSC would only apply to patients with LVO and may unnecessarily delay treatment in all others. Therefore, the predictive power of initial screening tools to identify patients with suspected LVO becomes of paramount importance. Various scoring systems have been developed to detect potential candidates to EVT. These scales must meet key criteria: rapid and simple to use, applicable to an unselected population with a suspected stroke, high interrater reliability, and high accuracy to avoid underdiagnosis (low false-negative rate; i.e., 1-sensitivity) and overdiagnosis that could overload the CSC (low false positive rate, i.e., 1-specificity). Finally, the scale must be validated and proven to improve patient outcomes (20). A recent observational study compared 13 validated prehospital scales and concluded that published cutoff scores to predict LVO in clinical settings were associated with high accuracy but also yielded a high false-negative rate (21).

Among them, one that showed high accuracy and a comparatively lower false-negative rate was the RACE scale. This scale is a simplification of the NIHSS scale, using only those items with a higher ability to predict the presence of LVO. The RACE scale evaluates 5 items: facial palsy, brachial paresis, crural paresis, oculocephalic deviation and aphasia/agnosia. Scores range from 0 to 9. A score > 4 allows the suspicion of a LVO with a sensitivity of 85% and specificity of 69% (22). The RACE scale was designed and validated in Catalonia with a prospective study that included 357 patients in 2011–2013. Therefore, in optimizing stroke systems of care, the prehospital assessment is a key factor to take into account.

## The stroke code system of catalonia as an example

**Figure 1:**
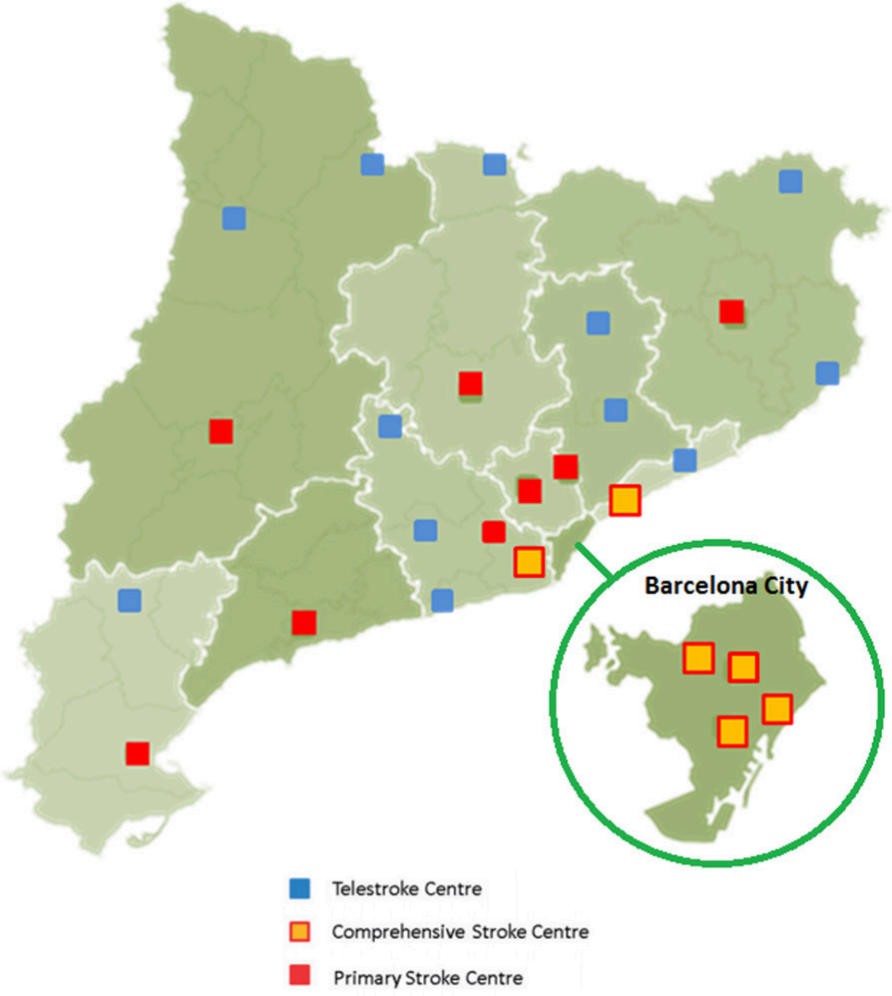
Geographical distribution of the Stroke Centres in Catalonia.

Catalonia has a population of 7.5 M and an organized and highly territorialized stroke system of care administered by the Stroke Programme, an organization created in 2004 by the Catalan Health Department. In 2006, the Stroke Programme implemented the Stroke Code System in 2006 to cover all the territory (23) (Figure 1). Current criteria for Stroke Code activation are clinical suspicion of acute stroke, less than 8 h from symptom onset (or unknown), and previous functional independence (Rankin 0–2) with no age limit (24, 25). Upon Stroke Code activation, the EMS coordinates patient transport to the nearest Stroke Centre (SC) according to predefined pathways. After initial recognition of stroke symptoms, the destination SC is pre-alerted by the EMS. The Stroke Code can be activated directly from EMS upon identification of a stroke patient in the field (60% of all Stroke Code activations) or at the Emergency Department of any hospital when patients arrive at the hospital by their own means. In recent years, our network of acute hospitals that are active in the stroke code system has grown to include 26 centers: (1) Twelve TSCs with capacity to deliver IVT via tele-consultation with a vascular neurologist who covers all tele-consultations from the 7 TSCs in the outer metropolitan area of Barcelona; the remaining 5 TSCs are located in the Catalan provinces and are usually covered by the neurologist on call at the nearest provincial PSC, with the central on-call service acting as a backup. (2) Eight PSCs with capacity to deliver IVT and admit patients to a certified Stroke Unit. (3) Six EVT-capable centers or CSCs, all of them located in the inner metropolitan area of Barcelona.

According to the Stroke Code protocol, patients with a suspected acute stroke are transferred to the closest SC or TSC in order to prioritize urgent expert evaluation and rapid IVT if indicated. This strategy is extremely effective and safe, increasing IVT treatment and reducing the time from symptom onset to IVT initiation; however, this decentralized model is associated with delayed EVT initiation and lower rates of EVT, compared to areas where patients are directly transferred to a CSC (26).

**Figure 2:**
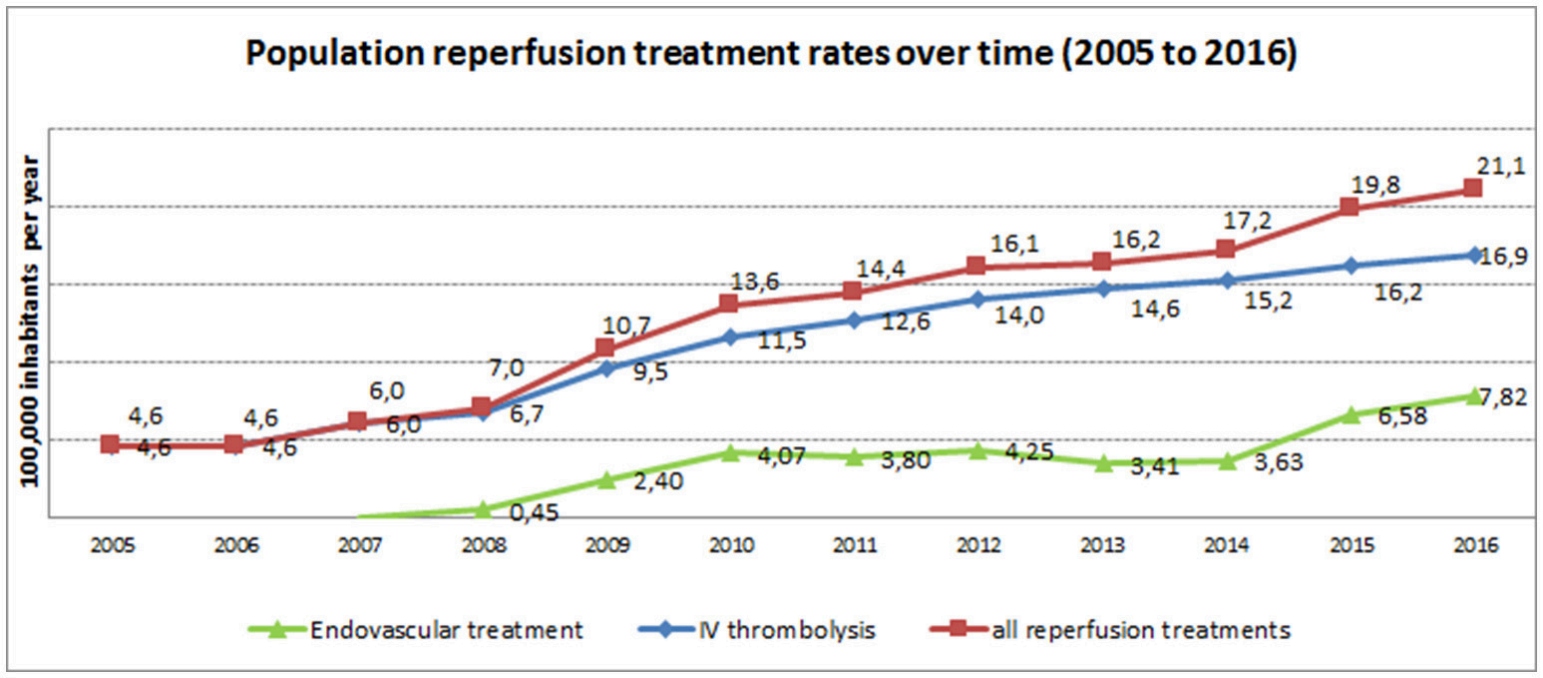
Temporal trends of population reperfusion therapies rates (2005–2016).

Another important approach that has proved useful to improve the Stroke Code System in Catalonia has been the analysis of big data. The vast amount of clinical data available constitutes a formidable resource for evaluation and research. Every day a large amount of stroke data (mostly unstructured or semi-structured) is generated in a great variety of sources that should be rapidly analyzed. One of these sources, population health registries, provides essential tools to obtain epidemiological data from patients with certain diseases, monitor therapies, and run audits of health services. This information is useful in the decision-making process when planning health policies in a given territory. Catalonia has a government-mandated, population-based registry (SONIIA) with external monitoring of data completeness, which assesses quality of reperfusion therapies delivered to ischemic stroke patients since 2011. Region-wide reperfusion treatment rates in Catalonia are among the highest in Europe (17.3% overall, 14.3 for IVT, and 6% for EVT in 2016, according to data from the Catalan Stroke Programme. Figure 2 shows the temporal trends of population reperfusion therapies since 2005. The increased number of reperfusion treatments is likely a result of improved organization of stroke care, as well as the less restrictive criteria used for eligibility to treatments, which have changed through the years.

A decrease in mean door-to-needle and door-to-groin-puncture times has also been observed over time, being currently 38 and 75 min, respectively (taking into account all types of centers).

To achieve all these goals, a high level of commitment of all SC teams is necessary. Due to the different levels of complexity in the SCs in Catalonia, stroke patients are treated by multidisciplinary teams of healthcare professionals. According to data from a 2017 survey by the Catalan Stroke Programme, neurologists with expertise in cerebrovascular diseases, neuroradiologists, neurosurgeons, vascular surgeons, rehabilitation specialists (including physiotherapists) and stroke unit nurses are the core of the stroke teams in Catalan CSCs. The eight PSCs have a lower number of specialists and the disciplines involved are more diverse. Finally, in the 12 telestroke centers, the main healthcare professionals involved in stroke care are emergency physicians (internists). We consider that CSCs have homogeneous and well-balanced specialist teams and that less variability should exist in the PSCs, with stroke teams including neurologists, neuroradiologists, rehabilitation specialists (including physiotherapists) and stroke nurses.

We would emphasize that population campaigns are crucial. In Catalonia, the RAPID campaign (similar to Act FAST in the US) was launched in 2008 to increase the general public's knowledge about how to detect a stroke.

The RACE scale was incorporated into the Stroke Code protocol in Catalonia in September 2014, after an online training program for EMS technicians and other EMS professionals. Currently, the RACE scale is evaluated by the EMS team, registered on the EMS database, and delivered to the receptor Stroke Centre in >85% (*n* = 5,073) of the Stroke Code activations (2016 data, Stroke Programme).

Based on a well-established stroke network of acute hospitals that work in close collaboration with the EMS and the existence of an exhaustive, population-based registry validated for research quality, the Stroke Code system of Catalonia offers the potential for innovative studies, such as the RACECAT trial.

## Ensuring universal access to optimal treatment. the big challenge: equity

Now that reperfusion therapies, specifically interventionism, have been shown to be beneficial in AIS patients with proximal LVO, stroke systems of care should be reorganized to provide acute treatment, including mechanical thrombectomy, in a timely and equitable manner. Recent studies, including one performed in Catalonia, have demonstrated that access to EVT from remote areas is limited in high-income countries (26) and geographic disparities in IVT use are increasing, showing a rural-urban inequality trend (27). In a rural area of North Carolina, researchers showed that re-organization of the stroke system of care (in that case, to pursuit official certification of the hospital) allowed patients to receive evaluation and treatment in a timely and efficient manner close to home (28). A recent systematic review aimed to determine the quality of existing stroke-care services in low- and middle-income countries and described great variability, with very low rates of reperfusion therapies (and mainly IVT) provided in large part by the private sector (29).

Though differences in every country must be examined independently, there is still room for improvement in stroke care organization, using strategies that have been proven to be economic, feasible and reproducible, such as EMS training in pre-hospital assessment and telestroke implementation in remote areas (30). Other initiatives-such as education of the general population (i.e., public health campaigns), involvement of public-sector healthcare personnel, hospital preparedness, and legislative and economic factors-are key to success in improving access to best-practice stroke care (31).

## Conclusions

Innovation in stroke systems of care is a key factor to achieve the main aim in stroke care: to build a national stroke plan capable of offering the best possible treatment to all patients eligible for reperfusion therapies. Necessary elements include a high level of organization, close cooperation with EMS (prehospital assessment), strong commitment of all stroke physicians at Stroke Centres, the availability of a disease-specific registry, and finally local government involvement to establish stroke care as a priority.

## Author contributions

RV-H and MG are the main authors. They have reviewed the bibliography and draft the manuscript. SA and MS-P have contributed revising the manuscript. AR and GG have contributed managing and analyzing the data presented of the Catalan Stroke Programme.

### Conflict of interest statement

The authors declare that the research was conducted in the absence of any commercial or financial relationships that could be construed as a potential conflict of interest.
